# The Anatomy of American Football: Evidence from 7 Years of NFL Game Data

**DOI:** 10.1371/journal.pone.0168716

**Published:** 2016-12-22

**Authors:** Konstantinos Pelechrinis, Evangelos Papalexakis

**Affiliations:** 1 School of Information Sciences, University of Pittsburgh, Pittsburgh, PA, United States of America; 2 Department of Computer Science and Engineering, University of California Riverside, Riverside, CA, United States of America; Mälardalen University, SWEDEN

## Abstract

How much does a fumble affect the probability of winning an American football game? How balanced should your offense be in order to increase the probability of winning by 10%? These are questions for which the coaching staff of National Football League teams have a clear qualitative answer. Turnovers are costly; turn the ball over several times and you will certainly lose. Nevertheless, what does “several” mean? How “certain” is certainly? In this study, we collected play-by-play data from the past 7 NFL seasons, i.e., 2009–2015, and we build a descriptive model for the probability of winning a game. Despite the fact that our model incorporates simple box score statistics, such as total offensive yards, number of turnovers etc., its overall cross-validation accuracy is 84%. Furthermore, we combine this descriptive model with a statistical bootstrap module to build FPM (short for Football Prediction Matchup) for predicting future match-ups. The contribution of FPM is pertinent to its simplicity and transparency, which however does not sacrifice the system’s performance. In particular, our evaluations indicate that our prediction engine performs on par with the current state-of-the-art systems (e.g., ESPN’s FPI and Microsoft’s Cortana). The latter are typically proprietary but based on their components described publicly they are significantly more complicated than FPM. Moreover, their proprietary nature does not allow for a head-to-head comparison in terms of the core elements of the systems but it should be evident that the features incorporated in FPM are able to capture a large percentage of the observed variance in NFL games.

## 1 Introduction

While American football is viewed mainly as a physical game—and it surely is—at the same time it is probably one of the most strategic sports games, a fact that makes it appealing even to an international crowd [1]. This has led to people analyzing the game with the use of data analytics methods and game theory. For instance, after the controversial last play call of Super Bowl XLIX the Economist [2] argued by utilizing appropriate data and game theory that this play was rational and not that bad after all.

The ability to analyze and collect large volumes of data has put forward a quantification-based approach in modeling and analyzing the success in various sports during the last few years. For example, pertinent to American football, Clark et al. [3] analyzed the factors that affect the success of a field goal kick and contrary to popular belief they did not identify any situational factor (e.g., regular vs post season, home vs away etc.) as being significant. In another direction Pfitzner et al. [4] and Warner [5] studied models and systems for determining a successful betting strategy for NFL games, while the authors in [6] show that the much-discussed off-field misconduct of NFL players does not affect a team’s performance. Furthermore, the spatial information collected from the RFID sensors on NFL players has been used to evaluate quarterbacks’ decision making ability [7], while efforts to assess the impact of individual offensive linemen on passing have been presented by Alamar and Weinstein-Gould [8]. Similarly, Correia et al. [9] analyzed the passing behavior of rugby players—the most similar sport to that of American football. They found that the time required to close the gap between the first attacker and the defense explained 64% of the variance found in pass duration and this can further yield information about future pass possibilities. Nevertheless, despite the availability of play data for American football and the proliferation of the sports analytics literature as well as the literature surrounding the NFL, there are only few—publicly open—studies that have focused on predicting a game’s outcome. Furthermore, some of the existing models make strong theoretical assumptions that are hard to verify (e.g., the team strength factors obeying to a first-order autoregressive process [10]). Close with our work, Cohea and Payton developed a logistic regression model to understand the factors affecting an NFL game outcome [11]. The benefit of our model as compared to the one presented by Cohea and Payton [11] is that the number of exploratory variables we are using is much smaller, making it easy for a fan to follow. Most importantly though we combine our model with statistical bootstrap in order to facilitate future game predictions (something that the model presented in [11] is not able to perform). Of course, predictive models for NFL games have been developed by major sports networks. For example ESPN has developed the Football Power Index, which is used to make probabilistic predictions for upcoming matchups [12]. Software companies have also developed their own models (e.g., Cortana from Microsoft [13]). Nevertheless, these models are proprietary and are not open to the public.

In this study we are first interested in providing a simple model that is able to quantify the impact of various factors on the probability of wining a game of American football. How much does a turnover affect a team’s probability of winning? Can you really win a game after having turned the ball over 5 times? While coaches and players know the qualitative answer to similar questions, the goal of our work is to provide a quantitative answer. For this purpose we use play-by-play data for the last seven seasons of the National Football League (i.e., between 2009 and 2015) and we extract specific team statistics for both the winning and losing teams. We then use the Bradley-Terry regression model [14, 15] to quantify the effect and statistical significance of each of these factors on the probability of wining a game of American football. This model is a descriptive one, i.e., it quantifies the impact of several factors on the success of an NFL team. Similar descriptive models can be useful to the coaching staff since they provide an *exact* quantification of the importance of each aspect of the game. They can also be helpful for the fans—especially the novice ones—for better understanding of the game. Evaluating the obtained model through cross validation provides an accuracy of 84% in predicting the winning team of a matchup.

The above descriptive model is able to provide accurate predictions when the features are known, i.e., when the performance of the two competing teams of a matchup is known. This can be helpful in post analysis of games by comparing the actual outcome of the game with the expected probability of winning the game for each team given their performance. For instance, one can identify “unexpected” wins from teams that *underperformed*. However, even more challenging, and one of the most intriguing tasks for professional sports analysts, is predicting the winners of the upcoming NFL matchups, which is the second objective of our work. This task can not be completed simply by the regression model that quantifies the impact of various factors on the probability of winning a game. As we will elaborate on in following sections the majority of the features in the developed model includes performance statistics (e.g., total offensive yards, number of interceptions etc.). Hence, the winner prediction problem involves also predicting the features—i.e., the performance of each team—themselves.

Predicting the upcoming performance of a team can be based on its past performance. A factor that makes this task particularly hard for American football is the small number of games during a season, which translates to high uncertainty. Using a central tendency metric—e.g., mean—is not able to fully capture the variability of the performance. To tackle this problem we propose to use statistical bootstrap. In brief, resampling with replacement the features from the past games of a team will allow us to simulate the matchup between the teams several times and obtain a set of winning probabilities that will allow us to predict the final winner of the game. Our approach, FPM, is shown to exhibit an accuracy of approximately 64% over the past 7 seasons, which is comparable to that of the state-of-the-art systems such as Microsoft’s Cortana and ESPN’s FPI. However, given FPM’s simplicity it should be treated as a baseline estimation. Simply put the output probability of our model can be thought of as an anchor value for the win probability. Further adjustments can be made using information about the specific matchup (i.e., roster, weather forecast etc.), hence, making it possible to significantly outperform existing proprietary systems. We further discuss this point in detail later in this work.

Our work complements the existing literature by contributing a descriptive and easily interpretable model for American football games. We further provide a prediction engine for upcoming matchups based on statistical bootstrap and the developed Bradley-Terry regression model. We would like to emphasize here that our regression model is rather simple and easy to implement. This, in fact, is one of our main contribution, since we demonstrate that such a simple and transparent approach is able to perform on par with state-of-the-art commercial tools for which due to their proprietary nature we have no telling of how complex they are. We view this as a first step towards exploring how we can maintain a simple and interpretable model that at the same time bears high predictive quality. In the rest of the study we present the data and methods that we used (see Section 2). We then present our regression model as well as FPM (see Section 3). We finally conclude and discuss the implications of our study (see Section 4).

## 2 Materials and Methods

In this section we will present the dataset we used to perform our analysis as well as the different methodological pieces of our analysis.

**NFL Dataset:** In order to perform our analysis we utilize a dataset collected from NFL’s Game Center for all the games (regular and post season) between the seasons 2009 and 2015. We access the data using the Python nflgame API [16]. The dataset includes detailed play-by-play information for every game that took place during these seasons. In total, we collected information for 1,792 regular season games and 77 play-off games. Given the small sample for the play-off games and in order to have an equal contribution in our dataset from all the teams we focus our analysis on the regular season games, even though play-off games are by themselves of interest in many perspectives.

**Bradley-Terry Model for Pairwise Comparisons:** The Bradley-Terry model is a method for ordering a given set of items based on their characteristics and understanding the impact of these characteristics on the ranking. In our case the set of items are the NFL teams and the output of the model for items *i* and *j* provides us essentially with the probability of team *i* (assuming with out loss of generality that *i* is the home team) winning team *j*. In particular, the Bradley-Terry model is described by [15]:

$$\mathrm{Pr}\left(T_{i}≻T_{j}|\pi_{i},\;\pi_{j}\right)=\frac{e^{\pi_{i}-\pi_{j}}}{1+e^{\pi_{i}-\pi_{j}}}$$ (1)

where *π*_*i*_ is the *ability* of team *i*. Given a set of team-specific explanatory variables **z**_*i*_, the difference in the ability of the teams *i* and *j* can be expressed as:

$$\sum_{r=1}^{k}\alpha_{r}\left(z_{ir}-z_{jr}\right)+U$$ (2)

where *U* ∼ *N*(0, *σ*^2^). The Bradley-Terry model is then a generalized linear model that can be used to predict the probability of team *i* winning team *j*. The above formulation does not explicitly treat possible ties between *i* and *j* (apart from the fact that if Pr(*T*_*i*_ ≻ *T*_*j*_|*π*_*i*_, *π*_*j*_) = 0.5 one can consider this as a tie between the two teams). However, in our case of NFL game prediction the probability of a game ending with a tie is extremely small and hence, we do not explicitly account for it. In particular, in our dataset there are only 3 regular season games that finished with a tie—post season games cannot end with a tie—which corresponds to a 0.1% probability. Nevertheless, there exist extensions of the Bradley-Terry model that are able to deal with ties if this is a highly probable outcome that needs to be modeled [15].

**Statistical Bootstrap:** In order to perform a game outcome prediction, we first need to forecast the performance of each of the contesting teams. However, we only have a (small) set of historic performance data for each team. Furthermore given that the performance of a team is not *stable*, using a measure of central tendency (e.g., sample mean) does not accurately capture the variability in the data. To overcome this problem we will rely on statistical bootstrap [17]. Statistical bootstrap is a robust method for estimating the unknown distribution of a population’s statistic when a sample of the population is known. The basic idea of the bootstrapping method is that in the absence of any other information about the population, the observed sample contains all the available information about the underlying distribution. Hence resampling with replacement is the best guide to what can be expected from the population distribution had the latter been available. By generating a large number of such resamples allows us to get a very accurate estimate of the required distribution. Furthermore, for data with dependencies (temporal or otherwise), appropriate block resampling retains any dependencies between data points [18]. We will utilize bootstrap in the design of FPM.

## 3 Results

### 3.1 Descriptive Model

In this part of our study we will present our descriptive generalized linear model. In particular, we build a Bradley-Terry model to understand the factors that impact the probability of a team winning an American football game. This model will be later used in our future matchup prediction engine, FPM, as we describe in Section 3.2.

Let us denote with *W*_*ij*_ the binary random variable that represents the event of home team *i* winning the game against visiting team *j*. *W*_*ij*_ = 1 if the home team wins the game and 0 otherwise. As aforementioned our model for *W*_*ij*_ will provide us with the probability of the home team winning the game given the set of input features, i.e., *y* = Pr(*W*_*ij*_ = 1|**z**). The input of this model is vector **z** that includes features that can potentially impact the probability of a team winning.

The features we use as the input for our model include:

**Total offensive yards differential:** This feature captures the difference between the home and visiting teams’ total yards (rushing and passing) produced by their offense in the game.

**Penalty yards differential:** This features captures the differential between the home and visiting teams’ total penalty yards in the game.

**Turnovers differential:** This feature captures the differential between the total turnovers produced by the teams (i.e., how many times the quarterback was intercepted, fumbles recovered by the opposing team and turns on downs).

**Possession time differential:** This feature captures the differential of the ball possession time between the home and visiting team.

**Passing-to-Rushing ratio *r* differential:** The passing-to-rushing ratio *r* for a team corresponds to the fraction of offensive yards gained by passing:

$$r=\frac{\#\text{of}\;\text{passing}\;\text{yards}}{\#\text{of}\;\text{total}\;\text{yards}}$$ (3)

This ratio captures the offense’s balance between rushing and passing. A perfectly balanced offense will have *r* = 0.5. We would like to emphasize here that *r* refers to the actual yardage produced and not to the passing/rushing attempts. The feature included in the model represents the differential between *r*_home_ and *r*_visiting_.

**Power ranking differential:** This is the current difference in rankings between the home and the visiting teams. A positive differential means that the home team is *stronger*, i.e., ranks higher, than its opponent. For the power ranking we utilize SportsNetRank [19], which uses a directed network that represents win-lose relationships between teams. SportsNetRank captures indirectly the schedule strength of a team and it has been shown to provide a better ranking for teams as compared to the simple win-loss percentage.

**Fig 1:**
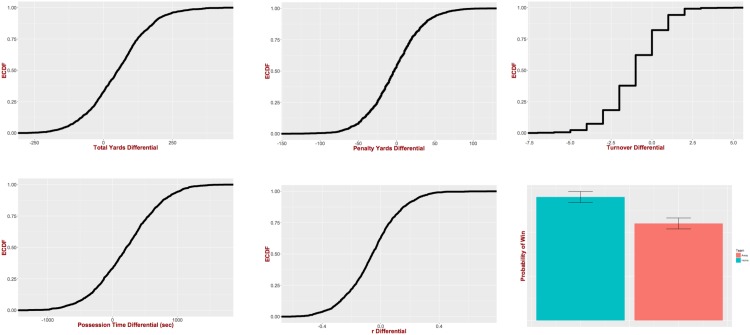
Empirical cumulative distribution function for the paired differences of each feature. Based on the Kolmogorov-Smirnov test the features’ ECDFs for the winning and losing teams are statistically different (at the significance level *α* = 0.01). The probability mass function for the home team advantage is also presented.

Before delving into the details of the descriptive model, we perform some basic analysis that compares the game statistics and metrics used for obtaining the features we include in our regression model. In particular, given a game statistic *s*_*i*_ (e.g., total offensive yards), we perform a paired comparison for this statistic between the winning and losing teams. In particular, for each continuous game statistic *s*_*i*_ we compare the pairs ($s_{i,j}^{+},\;s_{i,j}^{-}$) with a paired t-test, where $s_{i,j}^{+}$ ($s_{i,j}^{-}$) is the value of *s*_*i*_ for the winning (losing) team of the j^th^ game in our dataset. Table 1 depicts the results of the two-sided paired t-tests for our continuous statistics together with the home team *advantage* observed in our data. As we can see all the differences are significantly different than zero (at the significance level of *α* = 0.01). Fig 1 further presents the empirical cumulative distribution function (ECDF) for the paired differences for all the statistics as well as the probability mass function (PMF) for the distribution of the wins among home and visiting teams. For example, we can see that in only 20% of the games the winning team had more turnovers as compared to the losing team. We further perform the Kolmogorov-Smirnov test for the ECDFs of the considered statistics for the winning and losing teams. The tests reject the null hypothesis at the significance level of *α* = 0.01 for all cases, that is, the cumulative distribution of the features is statistically different for the winning and losing teams.

**Table 1:** Paired t-test for the considered game statistics. The difference represents $\bar{s_{i,j}^{+}}-\bar{s_{i,j}^{-}}$. Significance codes: ***: p < .001, **: p < .01, *: p < .05. The home team advantage is also presented.

Feature	Average paired difference
Total Yards	51.78 ***
Penalty Yards	-3.29*
Turnovers	-1.04***
Possession Time (sec)	211.79***
*r*	-0.06***
*Home Team Advantage*	56.03%±2.49%

Our basic data analysis above indicates that the distribution of the statistics considered is significantly different for the winning and losing teams. However, we are interested in understanding which of them are good explanatory variables of the probability of winning a game. To further delve into the details, we use our data to train the Bradley-Terry regression model and we obtain the results presented in Table 2. Note here that, as it might be evident from the aforementioned discussion, we do not explicitly incorporate a feature for distinguishing between the home and the visiting team. Nevertheless, the response variable is the probability of the home team winning, while the features capture the differential of the respective statistics between the home and road team (i.e., the difference is ordered). Therefore, the intercept essentially captures the home team advantage—or lack thereof depending on the sign and significance of the coefficient. In fact, setting all of the explanatory variables equal to zero provides us a response equal to Pr(*W_ij_*|**0**) = 0.555, which is equal to the home team advantage as discussed above. Furthermore, all of the coefficients—except the one for the possession time differential—are statistically significant. However, the impact of the various factors as captured by the magnitude of the coefficients range from weak to strong. For example, the number of total yards produced by the offense seem to have the weakest correlation with the probability of winning a game (i.e., *empty* yards). On the contrary committing turnovers quickly deteriorates the probability of winning the game and the same is true for an unbalanced offense. Finally, in S1 Text we present a standardized version of our model.

**Table 2:** Coefficients of our Bradley-Terry regression model for the random variable *W*_*ij*_. Significance codes: ***: p < .001, **: p < .01, *: p < .05.

Feature	Coefficient
Intercept	0.22**
Total Yards differential	0.01***
Penalty Yards differential	-0.02***
Turnovers differential	-1.05***
Possession Time differential	0.0001
*r* differential	-3.18***
Δ SportsNetRank	0.04***

While the direction of the effects for these variables are potentially intuitive for the coaching staff of NFL teams, the benefit of our quantifying approach is that it assigns specific magnitude to the importance of each factor. Clearly the conclusions drawn from the regression cannot and should not be treated as causal. Nevertheless, they provide a good understanding on what is correlated with winning games. For example, if a team wins the turnover battle by 1 it can expect to obtain an approximately 20% gain in the winning probability (all else being constant), while a 10-yard differential in the penalty yardage is correlated with just a 5% difference in the winning probability. Hence, while almost all of the factors considered are statistically significant, some of them appear to be much more important as captured by the corresponding coefficients and potential parts of the game a team could work on. Again, this descriptive model **does not provide a cause-effect relationship between the covariates considered and the probability of winning**.

**Fig 2:**
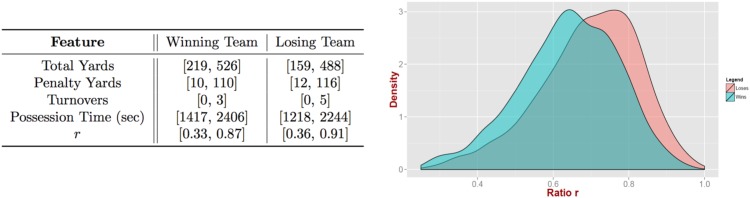
Model validity. Our model is trained within the range of input variable/statistics values on the left table. The figure on the right presents the probability density function for *r* for the winning and losing instances respectively.

**Fig 3:**
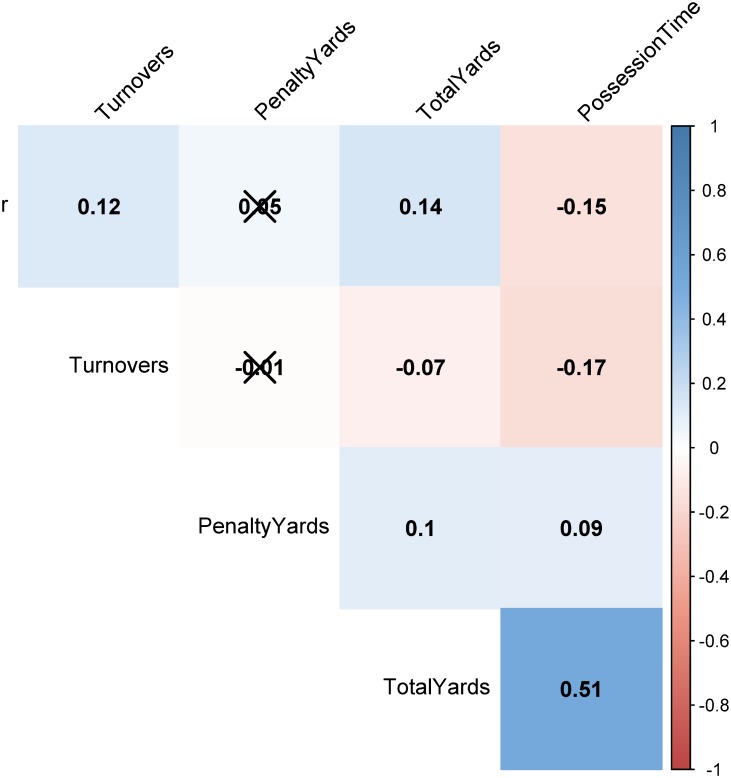
Correlograms. Correlations between the different variables considered for obtaining the features for FPM. Insignificant correlations are crossed out.

Before turning to the FPM predictive engine we would like to further emphasize and reflect on how one should interpret and use these results. For example, one could be tempted to focus on the feature with the coefficient that exhibits the maximum absolute magnitude, that is, the differential of ratio *r*, and conclude that calling only run plays will increase the probability of winning, since the negative differential with the opposing team will be maximized. However, this is clearly not true as every person with basic familiarity with American football knows. At the same time the regression model is not contradicting itself. What happens is that the model developed—similar to any data driven model—is *valid* only for the range of values that the input variables cover. Outside of this range, the generalized linear trend might still hold or not. For example, Fig 2 depicts the distribution of ratio *r* for the winning and losing teams. As we can see our data cover approximately the range *r* ∈ [0.3, 0.98] and the trend should only be considered valid within this range (and potentially within a small *ϵ* outside of this range). It is interesting also to observe that the mass of the distribution for the winning teams is concentrated around *r* ≈ 0.64, while it is larger for the losing teams (*r* ≈ 0.8). We also present at the same figure a table with the range that our features cover for both winning and losing teams. Furthermore, to reiterate, the regression model captures merely correlations (rather than cause-effect relations). Given that some of the statistics involved in the features are also correlated themselves (see Fig 3) and/or are result of situational football, makes it even harder to identify real causes. For instance, there appears to be a small but statistically significant negative correlation between ratio *r* and possession time. Furthermore, a typical tactic followed by teams leading in a game towards the end of the fourth quarter is to run the clock out by calling running plays. This can lead to a problem of reverse causality; a reduced ratio *r* for the leading team as compared to the counterfactual *r* expected had the team continued its original game-plan, which can artificially deflate the actual contribution of *r* differential on the probability of winning. Similarly, teams that are trailing in the score towards the end of the game will typically call plays involving long passes in order to cover more yardage faster. However, these plays are also more risky and will lead to turnovers more often, therefore, inflating the turnover differential feature. Nevertheless, this is always a problem when a field experiment cannot be designed and only observational data are available. While we cannot claim causal links between the covariates and the output variable, in what follows we present evidence that can *eliminate* the presence of reverse causality for the scenarios described above.

**Reverse Causality:** In what follows we examine the potential for reverse causality. To fast forward to our results, we do not find strong evidence for it. To reiterate, one of the problems with any model based on observational data is the direction of the effects captured by the model. For example, in our case teams that are ahead in the score towards the end of the game follow a “conservative” play call, that is, running the football more in order to minimize the probability of a turnover and more importantly use up valuable time on the clock. Hence, this can lead to a decreasing ratio *r*. Therefore, the negative coefficient for the *r* differential in our regression model might be capturing reverse causality/causation. Winning teams artificially decrease *r* due to conservative play calling at the end of the game. Similarly, teams that are behind in score towards the end of the game follow a more “risky” game plan and hence, this might lead to more turnovers (as compared to the other way around).

**Fig 4:**
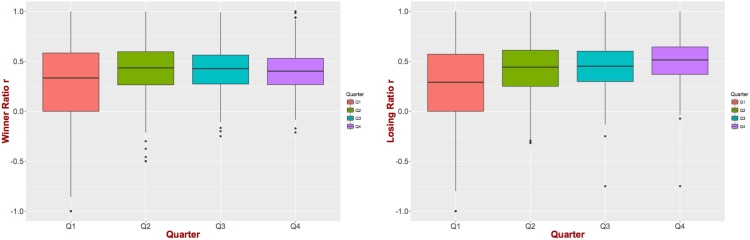
Evolution of *r* through the game. Ratio *r* is stable after the first quarter for both winning (left figure) and losing (right figure) teams, allowing us to reject the reverse causation hypothesis for *r*.

One possible way to explore whether this is the case is to examine how the values of these two statistics change over the course of the game. We begin with ratio *r*. If the reverse causation hypothesis were true, then the ratio *r* for the winning team of a game would have to reduce over the course of the game. In order to examine this hypothesis, we compute the ratio *r* at the end of each quarter for both the winning and losing teams. Fig 4 presents the results. As we can see during the first quarter there is a large variability for the value of *r* as one might have expected mainly due to the small number of drives. However, after the first quarter it seems that the value of *r* is stabilized. There is a slight decrease (increase) for the winning (losing) team during the fourth quarter but this change is not statistically significant. Therefore, we can more confidently reject the existence of reverse causality for ratio *r*.

**Fig 5:**
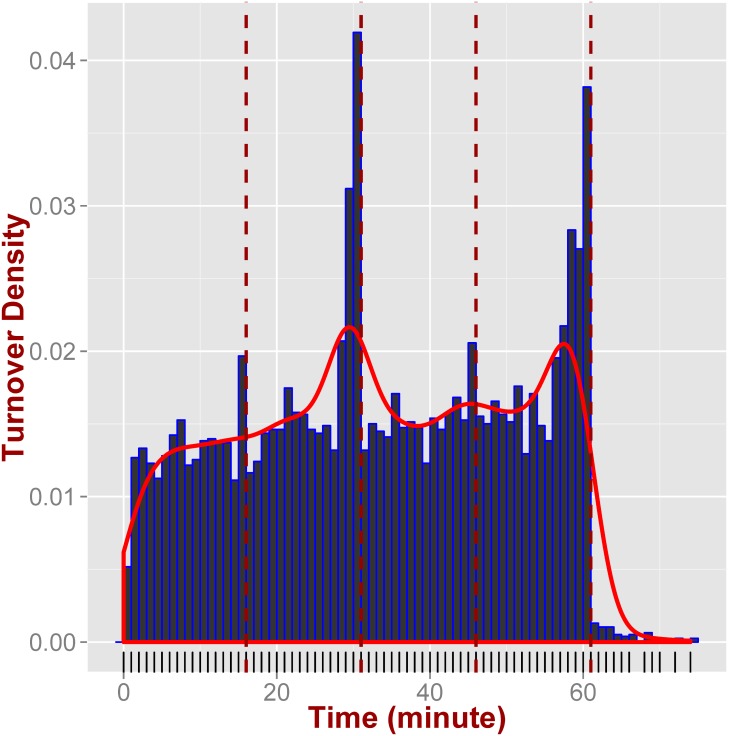
Temporal dynamics of turnovers. Turnovers spike towards the end of each quarter, with the highest density appearing during the two-minute warning.

We now focus our attention on the turnovers and the potential reverse causation with respect to this feature. In order to examine this hypothesis, we obtain from our data the time within the game (at the minute granularity) that turnovers were committed by the winning and losing teams. We then compare the paired difference for the turnover differential until the end of the third quarter for each game. Our results show that the winning teams commit fewer turnovers than their losing opponents by the end of the third quarter (*p*-value < 0.01), further supporting that avoiding turnovers will ultimately lead to a win. Of course, as we can see from Fig 5, there is a spike of turnovers towards the end of each half (and smaller spikes towards the end of each quarter). These spikes can be potentially explained from the urgency to score since either the drive will stop if the half ends or the game will be over respectively. However, regardless of the exact reasons for these spikes, the main point is that by committing turnovers, either early in the game (e.g., during the first three quarters) or late, the chances of winning the game are significantly reduced.

In conclusion, our model provides quantifiable and actionable insights but they need to be carefully interpreted when designing play actions based on it.

### 3.2 FPM Prediction Engine

We now turn our attention on how we can use the above model to predict the outcome of a future game. In a realistic setting, in order to be able to apply this regression model we will need to provide as an input the team statistics/features. This is by itself a separate prediction problem, namely, a team performance prediction problem. Hence, we begin by evaluating the prediction performance of the Bradley-Terry regression model itself using traditional machine learning evaluation methods. In particular, we evaluate the prediction accuracy of our model through cross validation. In this way we do not need to predict the value of the features but we explore the accuracy of the pure regression model. Using 10-fold cross validation we obtain an accuracy of **84.03%** ± **0.35%**. To reiterate this performance is conditional to the input features being known. From the inputs required for our model only two are known before the matchup, namely, the home team (which will allow us to formulate the response variable and the rest of the features appropriately) and the SportsNetRank differential. Thus, how can we predict the rest of the features, since in a realistic setting we will not know the performance of each team beforehand? Simply put, our FPM prediction engine will need to first estimate the two teams statistics/features (i.e., total yards, penalty yards, etc.) and then use the Bradley-Terry regression model to predict the winning team.

**Fig 6:**
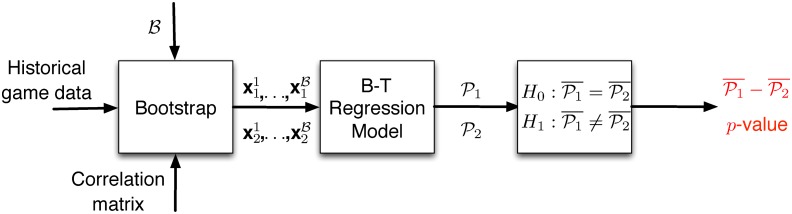
Football Matchup Prediction (FPM). The proposed prediction engine consists of 3 modules; a bootstrap module, a regression module and a statistical test module.

The most straightforward way for this task is to use historic game data from the current season and calculate descriptive statistics such as the mean or the median of each performance indicator of the teams and then compute the model’s features. The problem with this approach is that using a measure of central tendency does not accurately capture the variability in the teams’ performance. Therefore, we propose to utilize statistical bootstrap in order to resample with replacement B times the empirical distribution for each one of the team statistics from the observed sample. Multiple draws from the historic data will allow to properly characterize the input features of the model. For every resampling we can calculate the win probability for the home team using the Bradley-Terry regression model and ultimately obtain a confidence interval for the win probability for each of the teams. This essentially allows us to statistically compare the chances of each team winning the game. Fig 6 illustrates the components of FPM.

The first part of FPM obtains three inputs; (i) historical information for games of the current season, (ii) the number of bootstrap samples B to obtain and (iii) the correlation matrix between the features to be resampled. In particular, for every team *T* we have a matrix **M**_*T*_ each row of which represents a game in the current season, while the columns correspond to the five different statistics used in the features of the Bradley-Terry model. In the case of “simple” bootstrapping we would uniformly at random select for each performance statistic one row (i.e., one of the past performances of the team with respect to this feature) and hence, we would obtain a resampled vector $x_{T}^{i}$ that represents a potential performance for *T* given its past. However, there are two factors that we need to take into consideration. First, more recent games might be more representative of recent adjustments (or roster losses due to injuries) as compared to performances during the first weeks of the season. In order to control for this, instead of sampling the rows of **M**_*T*_ for every feature uniformly at random, we bias the sampling probabilities to favor the last *k* games of the team. Second, sampling the performance statistics independently can lead to vectors $x_{T}^{i}$ that do not exhibit the correlations that are present in the actual data. To reiterate Fig 3 represents the correlations between the different pairs of performance statistics. For example, as we can see the total yards and the possession time exhibit a medium to strong level of correlation. This means that when we sample for the total yards of the i^th^ bootstrap sample, we should not sample the possession time independently, but rather select the possession time from the same game/row of **M**_*T*_. This essentially mimics the block bootstrapping approach [18] used for time-series data to keep the dependencies between consecutive time-points. The rest of the correlations between the features are fairly weak (and some also insignificant) and hence, we proceed as normal with the rest of the statistics.

Once bootstrapping is completed its output is essentially a set of potential future performances for each team as captured through the obtained vectors. Simply put for each of the two competing teams we have bootstrapped vectors $x_{1}^{1},x_{1}^{2},\ldots ,x_{1}^{B}$ and $x_{2}^{1},x_{2}^{2},\ldots ,x_{2}^{B}$ respectively that capture the predicted game stats for the home and visiting team respectively. These vectors form the input for our regression model—in fact, the input for our model is $z^{j}=x_{1}^{j}-x_{2}^{1}$—which provides a set of winning probabilities for each team, i.e., $P_{1}=\left\{\mathrm{Pr}\left(W_{12}|z^{1}\right)\ldots ,\mathrm{Pr}\left(W_{12}|z^{B}\right)\right\}$, $P_{2}=\left\{1-P_{1}^{1},\ldots ,1-P_{1}^{B}\right\}$. Once we obtain these probability sets, we finally perform a hypothesis test to identify whether the two sets represent probabilities that are statistically different at a predefined significance level *α*:

$$H_{0}:\bar{P_{1}}=\bar{P_{2}}$$ (4)

$$H_{1}:\bar{P_{1}}\neq \bar{P_{2}}$$ (5)

If the null hypothesis is rejected, then the sign of the difference $\bar{P_{1}}-\bar{P_{2}}$ will inform us about the team that is most probable to win the matchup. If the null hypothesis is not rejected, then we can predict a tie. It should be evident that our predictive engine cannot be applied during the first week of the season, while the weekly variability of teams’ performance can be fully exploited in later parts in the season. Considering that, we set *k* = 5 in the current version of FPM and for each NFL season we start our predictions from Week 6. When focusing on a specific NFL season for predicting the game outcomes, we train our model using data from the rest of the seasons. Note here that the coefficients presented in Table 2 are obtained using all 7 years worth of data. When training the model using all the possible subsets of 6 seasons the obtained coefficients differ but not in any meaningful way. We further set B=1,000 and *α* = 0.05. The overall accuracy of FPM is **63.4%** with a **standard error of 1.3%**. This performance is not statistically different than the accuracy of the current state-of-the-art NFL prediction systems. For example, Microsoft’s Cortana system exhibited an accuracy of approximately 64.5% [20] during the last two seasons that it has been operating. Similarly, the prediction accuracy of ESPN’s FPI is 63% as well [12]. Furthermore, we randomly sampled forecasts of sports analysts from major networks (ESPN, NFL network, CBS and FOX sports) [21]. Our predictions were on average better than approximately 60% of the expert predictions.

Delving more into the evaluation of our predictive engine we present the accuracy for each season in Table 3. We also provide the accuracy of a baseline system, where the winner of a game is predicted to be the team with the better running win-loss percentage through the current week. If two teams have the same win-loss percentage the home team is chosen as the winner since there is a slight winning bias for the home team as we have seen earlier. Note here that the baseline is very similar to the way that the league ranks the teams and decides on who will qualify for the playoffs (excluding our tie-breaker process and the league’s rules with respect to the divisions). As we can see our predictive engine improves over the baseline by approximately 9%.

**Table 3:** Prediction accuracy. FPM outperforms the baseline prediction based on win-loss standings every season in our dataset. The overall accuracy of our system is 63.4%.

Year	Regression	Baseline
2009	0.66	0.57
2010	0.60	0.5
2011	0.68	0.58
2012	0.72	0.56
2013	0.55	0.5
2014	0.66	0.56
2015	0.57	0.53

**Fig 7:**
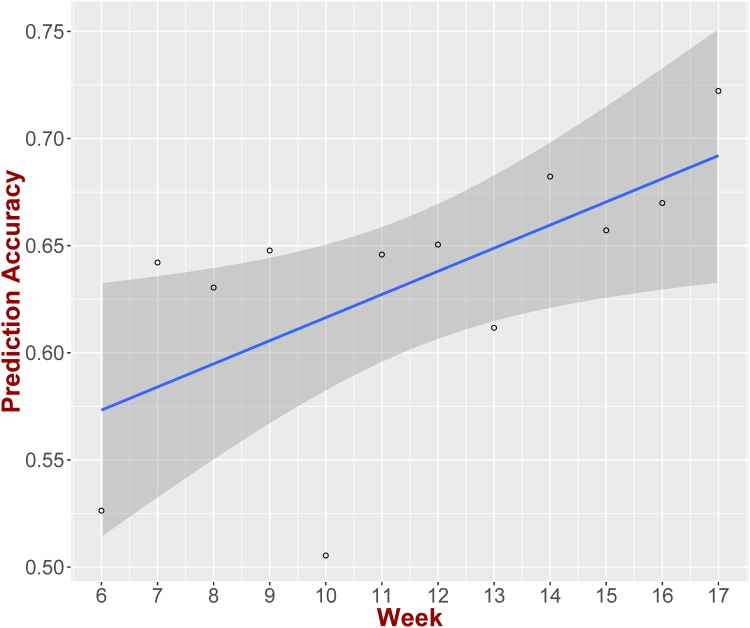
Prediction accuracy VS week. During the last part of the season the bootstrap engine can exploit the variability of a team’s performance better, hence, providing better prediction accuracy. The linear trend slope is 0.01 (p-value<0.05, *R*^2^ = 0.41).

One of the reasons we utilize bootstrap in our prediction system is to better capture the variability of the teams’ performances. As one might expect this variability is better revealed as the season progresses. During a stretch of few games it is highly probable to have a team over/under-perform [22]. Hence, the bootstrap module during the beginning of the season might not perform as accurately as during the end of the season. In order to examine this we calculate the accuracy of our prediction system focusing on games that took place during specific weeks in every season. Fig 7 presents our results, where we see that there is an increasing trend as the season progresses.

**Fig 8:**
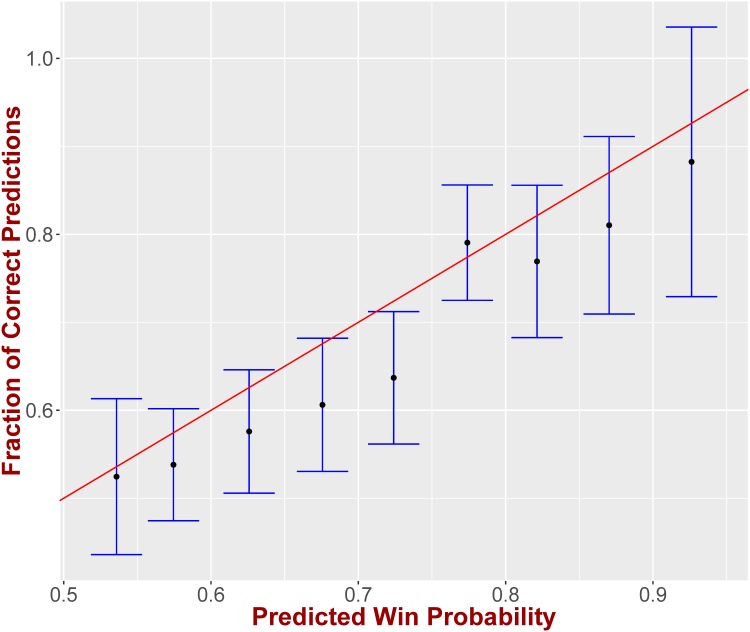
Probability Accuracy. The win probability provided by our model is in alignment with the fraction of the games won by the favorite for the corresponding win probability.

Finally, we examine the accuracy of FPM’s predicted probabilities. In order to evaluate this we would ideally want to have the game played several times. If the favorite team were given a 75% probability of winning, then if the game was played 100 times we would expect the favorite to win 75 of them. However, we cannot have the game play out more than once and hence in order to evaluate the accuracy of the probabilities we will use all the games in our dataset. In particular, if the predicted probabilities were accurate, when considering all the games where the favorite was predicted to win with a probability of *x*%, then the favorite should have won in *x*% of these games. Given the continuous nature of the probabilities we quantize them into groups that cover a 5% probability range (with only exception being the range (90%, 100%], since there are very few games in the corresponding sub-groups). Fig 8 presents on the y-axis the fraction of games where the predicted favorite team won, while the x-axis corresponds to the predicted probability of win for the favorite. As we can see the data points—when considering their 95% confidence intervals—fall on the *y* = *x* axis, which translates to an accurate probability inference. The corresponding linear regression provides a slope with a 95% confidence interval of [0.76, 1.16] (*R*^2^ = 0.94), which essentially means that we cannot reject the null hypothesis that our data fall on the line *y* = *x* where the slope is equal to 1.

## 4 Discussion and Conclusions

In this work we collected and analyzed 7 seasons of NFL play-by-play game data. In particular, we build a descriptive model for the probability of the home team winning an NFL game, which we combine with statistical bootstrap to provide a future matchup prediction (FPM) engine. Our results indicate that our Bradley-Terry regression model exhibits an 84% accuracy, while FPM—despite its overall simplicity—has a performance comparable to that of the current state-of-the-art (proprietary) NFL prediction systems. This simplicity allows for further improvements by considering the output of our prediction system simply as an *anchoring* probability [23] from which an expert can adjust his/her prediction using game-specific information. Our system is agnostic to game-day decisions (e.g., roster decisions, etc.) and hence, there is room for improvement by appropriately adjusting the bootstrap module. The latter is considerably flexible. For instance, one of the limitations of our system is that currently it does not incorporate any information for the schedule strength of teams (apart from the indirect consideration during the calculation of SportsNetRank). Simply put, when performing the bootstrap for the future performance of team *T* we just extrapolate from the older performance of *T* without considering the strength of the teams that it has played against. Teams might have put up a lot of offensive yards just because they have played with teams with poor defense. For example, in the 2014 NFL season the winner of NFC South (Carolina Panthers) had a season record less than 0.5. This means that Carolina had faced mainly teams with a losing record and hence, the corresponding team statistics might have been inflated. However, our engine is easily adoptable to account for this. In particular, let us assume that we want to estimate the performance of team *T*_1_ against *T*_2_. In order to account for the strength of *T*_2_, we can bias the resampling probabilities based on the propensity score [24] of the set of teams T that *T*_1_ has faced in the past. The propensity score is mainly used as a quasi-experimental technique for matching a treated sample with an untreated observational set based on a number of observable confounders. In our setting, the propensity score *γ*_*i*_ for $T_{i}\in T$ will essentially provide us with a “similarity” measure of *T*_*i*_ with *T*_2_. Consequently, the resampling probability for *T*_*i*_ will be proportional to *γ*_*i*_. Including defense-oriented features in the propensity score matching will allow us to perform a more balanced prediction—i.e., consider both offense and defense—since defensive attributes are currently underrepresented in the Bradley-Terry regression model. This has the potential to significantly improve the prediction performance of our engine.

Finally, the models themselves can be helpful to many different involved entities associated with the sport. For example, it can facilitate better understanding of the game by novice fans. The impact and importance of ratio *r* will allow the newcoming fans to appreciate the running game. Similarly, agents and players can use knowledge obtained by similar models for negotiating purposes. It is well-known that running backs are among the least paid players in an NFL roster for a number of reasons (e.g., high risk of serious injuries etc.). Nevertheless, they are extremely important for the success of a team as our model indicates. Moreover, our descriptive regression model can be used by media personnel for a post-game analysis. For instance, “surprising” wins can be identified, while critical parts of the game that led to the final results can also be pinpointed.

## Supporting Information

S1 Text Standardized FPM. (PDF)
