# Supplementary material for: The Anatomy of American Football: Evidence from 7 Years of NFL Game Data
Source: PLoS One. 2016 Dec 22;11(12):e0168716. doi: 10.1371/journal.pone.0168716 (PMC5179002; doi:10.1371/journal.pone.0168716)
Supplement: S1 Text — (PDF) [file pone.0168716.s001.pdf]

S1 Text. Standardized `FPM`**.** For easier interpretation and comparison between the different covariates of the model we present a standardized version of `FPM` (Table A). In particular, for each feature of the model we subtract the corresponding mean and divide with the standard deviation. In this case, all the coefficients correspond to an one standard deviation change in the covariate and hence, a straight-forward comparison of the magnitude of the coefficient across covariates is possible.

**Feature**	**Coefficient**
Intercept	0.50**
Total Yards differential	1.82***
Penalty Yards differential	-0.83***
Turnovers differential	-2.08***
Possession Time differential	-0.07
r differential	-0.63***
Δ `SportsNetRank`	0.55***

Table A. Standardized coefficients of our Bradley-Terry regression model for the random variable W_{ij}. Significance codes: *** : p < .001, ** : p < .01, * : p < .05.
